# Progressive Bulbar Weakness in an Older Male With Suspected Double-Seronegative Myasthenia Gravis Culminating in Myasthenic Crisis: A Case Report

**DOI:** 10.7759/cureus.109753

**Published:** 2026-05-27

**Authors:** Elina Kurkurina, Ravinderjeet K Puar, Jahnavi Mundluru, Mariam Farhan

**Affiliations:** 1 Internal Medicine, Frank H. Netter MD School of Medicine, North Haven, USA; 2 Internal Medicine, St. Vincent's Medical Center, Bridgeport, USA

**Keywords:** bulbar muscle weakness, double-seronegative myasthenia gravis, intravenous immunoglobulin, myasthenic crisis, plasmapheresis

## Abstract

Myasthenia gravis is a rare autoimmune disorder affecting the neuromuscular junction, causing muscle weakness that worsens with use and improves with rest. While it is typically diagnosed through antibody tests or electrophysiological testing, a smaller subset of the population is seronegative or may have inconclusive electrophysiological results. In emergent presentations, seronegative myasthenia gravis is a challenge to diagnose clinically, making it imperative to identify it early to ensure proper management and treatment. Here, we present the case of an older male with a complex medical history and suspected seronegative myasthenia gravis who through the course of his admission experienced myasthenic crisis necessitating treatment with both intravenous immunoglobulin and plasmapheresis.

## Introduction

Myasthenia gravis is a rare autoimmune disorder caused by antibodies targeting postsynaptic acetylcholine receptors or receptor-associated proteins at the neuromuscular junction, resulting in impaired neuromuscular transmission [1]. Women are typically affected earlier in life, while men are usually affected later [1]. The most common initial presentation is ocular muscle weakness characterized by ptosis or diplopia. Progression of the disease often involves bulbar, limb, and respiratory muscle weakness that worsens with use and improves with rest [1].

Myasthenia gravis is typically diagnosed through an anti-acetylcholine receptor antibody test. However, approximately 10% to 20% of patients with myasthenia gravis are seronegative and do not have detectable anti-acetylcholine receptor antibodies. These patients should instead be assessed for anti-Muscle-Specific Kinase (MuSK) antibodies or anti-low density lipoprotein receptor-related protein 4 (LRP4) antibodies [2-4]. A small subset of the population is double seronegative and does not have detectable anti-acetylcholine receptor antibodies or anti-MuSK antibodies, but may be anti-LRP4 positive [4,5]. Patients with seronegative myasthenia gravis are less likely to have characteristic thymomas [3,6]. Initial treatment focuses on acetylcholinesterase inhibitors and corticosteroids, with variable response [7]. 

Approximately 15% of myasthenia gravis cases have a bulbar presentation including dysarthria and dysphagia [1,8,9]. Exacerbation of myasthenic symptoms and fatigability of bulbar muscles can lead to myasthenic crisis presenting as an acute respiratory insufficiency requiring intensive medical care [3,10]. Here, we present the case of a 74-year-old male patient who presented with suspected seronegative myasthenia gravis and through the course of his admission developed bulbar weakness leading to a myasthenic crisis, necessitating treatment with intravenous (IV) immunoglobulin and plasmapheresis to improve persistent dysphagia.

## Case presentation

A 74-year-old African American male with a complex past medical history, including hypertension, insulin dependent diabetes mellitus, coronary artery disease treated with drug eluding stents complicated by in stent thrombosis, heart failure with reduced ejection fraction of 34%, peripheral vascular disease with left lower extremity ischemia treated with thrombectomy and stents complicated by compartment syndrome requiring emergent fasciectomy, benign prostatic hyperplasia treated with transurethral resection of the prostate complicated by ureteral stricture requiring self-catheterization, stage IIIa chronic kidney disease, and multiple small left middle cerebral artery strokes presented to the emergency department with dyspnea and bilateral lower extremity edema.

**Figure 1:**
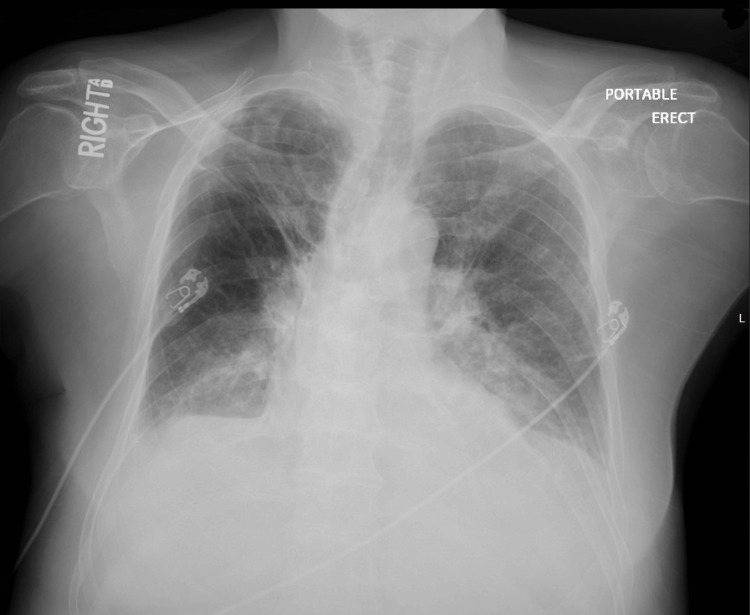
X-ray at the initial presentation to the emergency department

In the emergency department, his blood pressure was 220/128 mmHg (reference: <120/80 mmHg), heart rate was 111 beats per minute (reference range: 60-100 beats per minute), temperature was 36.7 °C, respiratory rate was 22 breaths per minute (reference range: 12-20 breaths per minute), and pulse oximetry was 100% on 4 L of O2 via nasal canula. On exam, he was tachycardic and fluid overloaded with rhonchi, rales, and bilateral lower extremity edema. His laboratory assessment revealed a potassium of 7.3 mmol/L (reference range: 3.4 - 4.5 mmol/L), creatinine of 2.2 mg/dL (baseline 1.5 mg/dL, reference range: 0.7 - 1.3 mg/dL), and B-type natriuretic peptide (BNP) of 3,186 pg/mL (reference range: 0 - 99 pg/mL). The chest X-ray (Figure 1) showed diffuse increased broncho-vascular markings, most pronounced in the perihilar and infrahilar distribution, left lower lobe opacities and blunting of both costophrenic angles.

The patient was admitted for management of heart failure exacerbation, hypertensive urgency, hyperkalemia, and cardiorenal acute kidney injury and started on IV furosemide.

Four months prior to this admission, he underwent outpatient myasthenia gravis autoimmune testing for the evaluation of weakness and dysphagia. While awaiting results from a myasthenia gravis panel, he was started on 60 mg of pyridostigmine three times daily and 20 mg of prednisone once daily. He showed an improvement on this regimen. The panel subsequently revealed that his acetylcholine receptor binding antibodies were <0.30 nmol/L (negative less than or equal to 30 nmol/L), acetylcholine receptor blocking antibodies were <15% inhibition (negative less than 15% inhibition), and acetylcholine receptor modulating antibodies were <1% inhibition (negative less than 32% inhibition), raising suspicion for seronegative myasthenia gravis. His striated muscle antibody screen was also negative. He did not undergo single fiber electromyography at that time. He reported adherence to his medication regimen but could not recall the names of medications, doses, or frequency of administration. 

**Figure 2:**
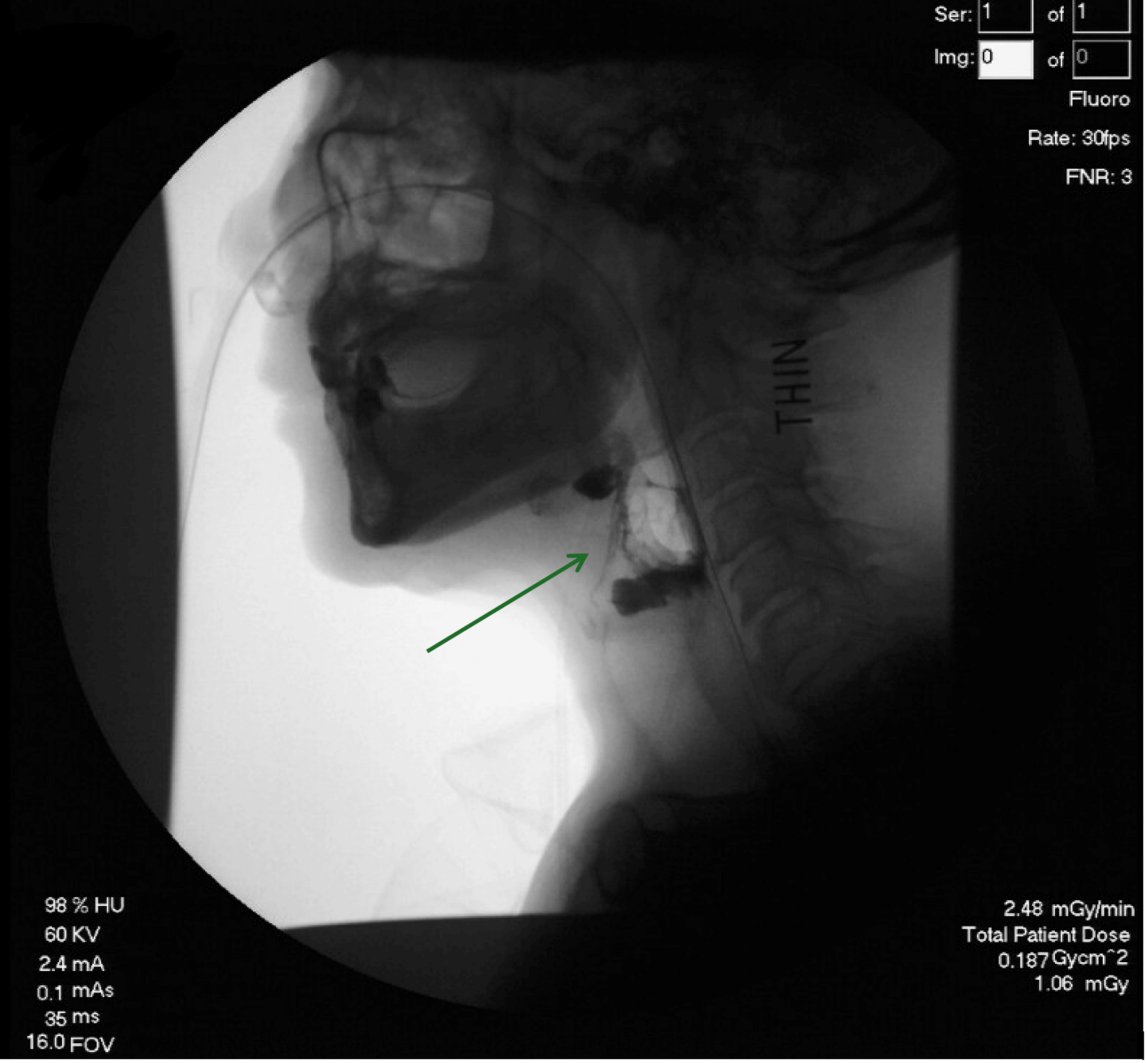
Initial modified barium swallow showing reduced pharyngeal function

On day four of hospitalization, the patient developed progressively worsening dyspnea. Physical exam findings progressed from mild bibasilar crackles that were treated with IV furosemide to prolonged expiration, gurgling sounds from the upper airway, bilateral inspiratory crackles extending up to mid lung fields, diminished breath sounds over the bilateral bases, and a weak cough. His speech became quieter and weaker. A modified barium swallow test (Figure 2) showed diffusely reduced function of pharyngeal physiology, and a nasogastric tube was placed.

His arterial blood gases showed a pH of 7.34 (reference range: 7.35-7.45), partial pressure of carbon dioxide of 41 mmHg (reference range: 35-45 mmHg), partial pressure of oxygen of 71 mmHg (reference range: 80-100 mmHg), and a bicarbonate of 22 mmol/L (reference range: 22-26 mmol/L). Due to a concern for rapid deterioration, he was transferred to the Intensive Care Unit (ICU) for management of suspected myasthenic crisis. He was tested for anti-MuSK antibodies, which were not detected, increasing suspicion for double seronegative myasthenia gravis. 

**Figure 3:**
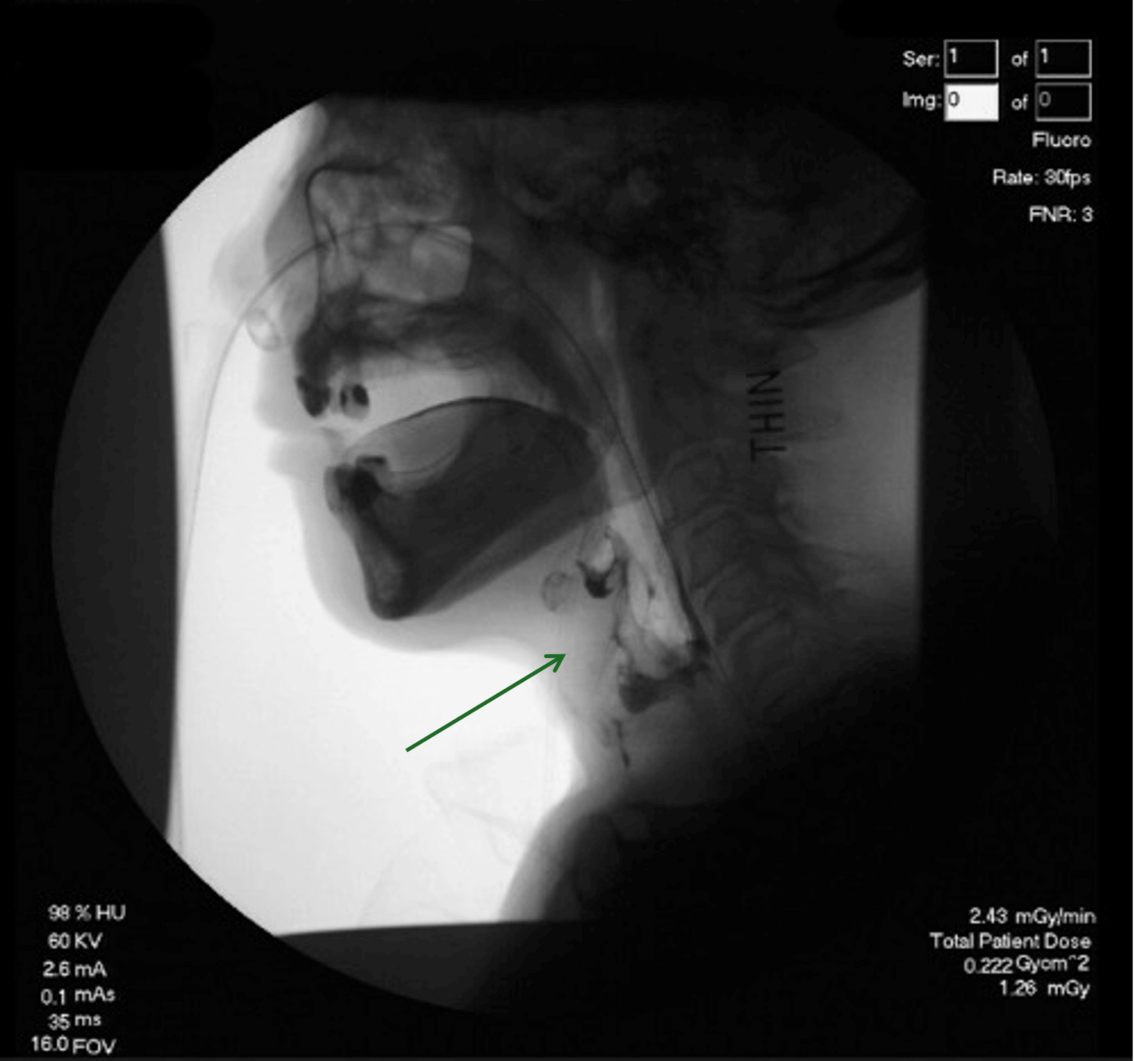
Modified barium swallow showing residual thin fluid, indicating dysphagia This was after completing five days of intravenous immunoglobulin.

In the ICU, he was treated with supplemental oxygen, IV furosemide, and started on five days of IV immunoglobulin. He did not require intubation and his NIF improved to - 40 with medical management. His neurological exam showed he was awake, alert and oriented to name, place, month, date, and year. His speech was fluent although nasal/guttural, with no dysarthria. He was able to name objects and repeat phrases. His comprehension was intact and he followed commands. His pupils were small, equal, and reactive to light with intact extraocular movements. He had normal facial sensation. His face was symmetric and tongue was midline. He had normal bulk and tone, 5/5 strength throughout, including neck flexion and extension. He was able to perform the finger to nose task with no dysmetria. Sensation was intact to light touch. He was initially transferred to the progressive care unit after one day of ICU management and then to the inpatient unit with Speech and Language Pathology evaluations and daily monitoring of his NIF. Upon completion of five days of IV immunoglobulin, he had persistent dysphagia (Figure 3), hypophonia, and a weak cough.

**Figure 4:**
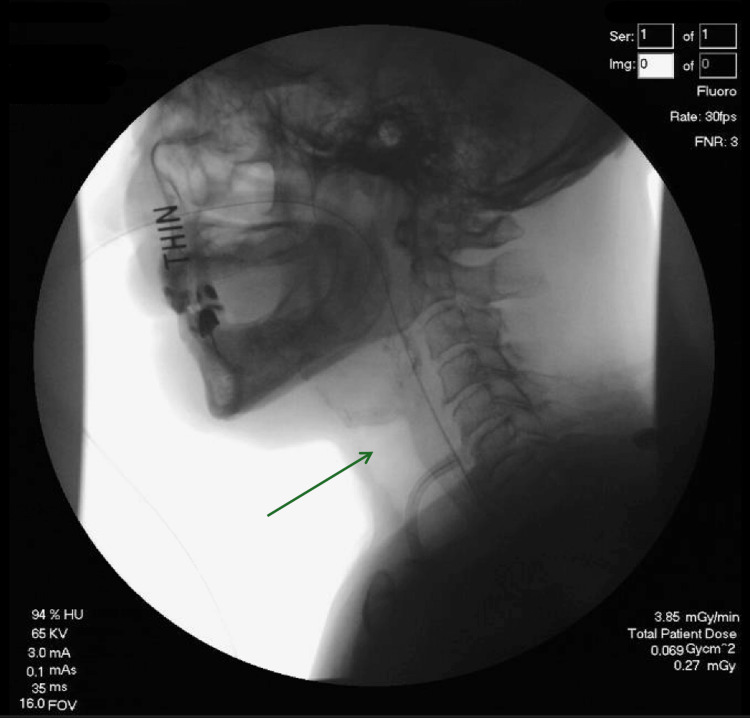
Modified barium swallow showing no residual fluid, indicating improvement in dysphagia This was after completing five sessions of plasmapheresis.

Although his dyspnea improved, he did not show clinical improvement in dysphagia after five days of monitoring and was subsequently started on five sessions of plasmapheresis. By his fourth session, he showed steady improvement in cough strength and in his NIF, which was now at - 60. Upon completion of five sessions of plasmapheresis, his dysphagia had significantly improved (Figure 4).

His NIF values ranged from - 40 to - 60 on subsequent days of his admission. He was discharged home with services and a neurology follow-up referral. At the time of discharge, he was continued on 50 mg of prednisone daily and 60 mg of pyridostigmine three times a day. Along with his other medications, these medications were packaged into blister packs to improve ease of administration and adherence.

## Discussion

This case highlights the importance of monitoring for respiratory weakness in patients with confirmed or suspected myasthenia gravis. In emergent presentations, seronegative myasthenia gravis is a challenging diagnosis [3,11]. Diagnosis often includes an anti-acetylcholine receptor antibody assay, repetitive nerve stimulation, or single fiber electromyography with sensitivities of 73%, 77%, and 92%, respectively [12]. Older adults who present with bulbar symptoms are typically worked up for cerebrovascular events [13] or motor neuron disease such as amyotrophic lateral sclerosis [9]. These conditions may have similar presentations, including bulbar muscle involvement leading to dysarthria or dysphagia. However, unlike myasthenia gravis, these conditions often do not improve with the administration of acetylcholinesterase inhibitors. For patients with negative workups for cerebrovascular events or motor neuron diseases, myasthenia gravis should be on the differential, and these patients should be closely monitored for signs of respiratory distress that can quickly deteriorate.

NIF, also referred to as maximal inspiratory pressure, measures the strength of the diaphragm and can be used to monitor for respiratory decline. Patients are instructed to breathe in as hard as possible through a spirometer or mask that measures their inspiratory force over 1.5 seconds. The mean maximal inspiratory pressure varies by age and sex. Women tend to have lower mean pressures than men, and the mean pressure decreases with age across the sexes [14]. Elective intubation should be considered for patients with NIF values in the 0 to - 30 range, as it indicates profound respiratory weakness. While intubation was not necessary for our patient, patients with myasthenia gravis should be induced with non-depolarizing agents, such as rocuronium or vecuronium. Patients with myasthenia gravis have increased resistance to succinylcholine and increased sensitivity to nondepolarizing agents [15].

For patients who do not improve on IV immunoglobulin, plasmapheresis should be considered. The timing of plasmapheresis following IV immunoglobulin is complicated, as rapid initiation of plasmapheresis counters the effect of the administered IV immunoglobulin. In our case, the patient was monitored for clinical improvement over the course of several days. While his dyspnea improved on IV immunoglobulin, his persistent dysphagia made him an unsafe discharge. He underwent five plasmapheresis sessions. Data show that there is no added clinical benefit to extending the number of plasmapheresis sessions beyond five [16]. Upon completion of these sessions, the patient’s dysphagia improved, and he was able to safely tolerate oral intake.

Lastly, there are unique challenges associated with management of myasthenia gravis in older adults [17]. While acetylcholinesterase inhibitors are typically well tolerated, they are associated with gastrointestinal distress, urinary frequency, urgency, and incontinence, as well as bradycardia which may increase fall risk among patients with limited mobility rushing to the toilet [17]. Similarly, immunosuppression from corticosteroids presents unique risks for older adults undergoing immunosenescence [17]. Corticosteroids further contribute to osteoporosis, osteonecrosis, dyslipidemia, hypertension, diabetes, heart failure, ischemic heart disease, and cerebrovascular disease exacerbating preexisting comorbidities [17]. As individuals continue to live longer, it is important to identify and manage myasthenia gravis safely among aging populations.

## Conclusions

Bulbar muscle weakness is a rare initial presentation of myasthenia gravis that is often missed or misdiagnosed in older patients, especially those who are seronegative. Patients with known or suspected myasthenia gravis should be carefully monitored for respiratory decline throughout the course of their admission, as stress and illness can precipitate a myasthenic crisis. A NIF is a non-invasive measure of diaphragm strength that can be used in the inpatient setting. Patients can be treated with IV immunoglobulin and plasmapheresis, although the timing of plasmapheresis following IV immunoglobulin is a nuanced clinical decision.
